# Potential strategies for sustainably financing mental health care in Uganda

**DOI:** 10.1186/s13033-018-0252-9

**Published:** 2018-12-05

**Authors:** J. Ssebunnya, S. Kangere, J. Mugisha, S. Docrat, D. Chisholm, C. Lund, F. Kigozi

**Affiliations:** 1 Butabika National Referral and Teaching Mental Hospital, Kampala, Uganda; 2 Alan J Flisher Centre for Public Mental Health, Department of Psychiatry and Mental Health, University of Cape Town, Cape Town, South Africa; 3 Department of Mental Health and Substance Abuse, World Health Organization, Geneva, Switzerland; 4 Institute of Psychiatry, Psychology and Neuroscience, Health Services and Population Research Department, Centre for Global Mental Health, King’s College London, London, UK

**Keywords:** Mental health, Financing, Uganda, Insurance, LAMICs

## Abstract

**Background:**

In spite of the pronounced adverse economic consequences of mental, neurological, and substance use disorders on households in most low- and middle-income countries, service coverage and financial protection for these families is very limited. The aim of this study was to generate potential strategies for sustainably financing mental health care in Uganda in an effort to move towards increased financial protection and service coverage for these families.

**Methods:**

The process of identifying potential strategies for sustainably financing mental health care in Uganda was guided by an analytical framework developed by the Emerging Mental health systems in low and middle income countries (EMERALD project). Data were collected through a situational analysis (public health burden assessment, health system assessment, macro fiscal assessment) and eight key informant interviews with selected stakeholders from sectors including health, finance and civil society. The situational analysis provided contextualization for the strategies, and was complimented by views from key informant interviews.

**Results:**

Findings indicate that the following strategies have the greatest potential for moving towards more equitable and sustainable mental health financing in the Uganda context: implementing National Health Insurance Scheme; shifting to Results Based Financing; decentralizing mental health services that can be provided at community level; and continued advocacy with decision makers with evidence through research.

**Conclusion:**

Although several options were identified for sustainably financing mental health care in Uganda, the National Health Insurance Scheme seemed the most viable option. However, for the scheme to be effective, there is need for scale up to community health facilities and implementation in a manner that explicitly includes community level facilities.

## Introduction

There is strong international consensus for integration of mental health care into primary care as the most viable way to narrow the large treatment gap for Mental, Neurological and Substance Abuse (MNS) disorders in low- and middle-income countries (LAMICs) [1]. While evidence of the feasibility and efficacy of intervention programs for integration is strong [2, 3], decision makers need data on the costs and cost-effectiveness of these programs to deliver successful and sustainable scaled-up mental health care [4].

Financing is a fundamental building block on which the other critical aspects of any system rest.

Similarly, adequate and sustained financing is a critical factor for translation of plans into action, towards realization of a viable health system. In countries without well-articulated mental health systems, ensuring that mental health financing is an integral component of general health financing has been noted to be a more viable option [5].

With the global movement towards Universal Health Coverage (UHC) gaining momentum, Uganda’s proposed national health insurance policy, is one of the policies relevant to UHC, a powerful way to reducing social disorder and conflicts through promoting equality in health access. The Health Sector Development Plan (HSDP) 2016–2020 emphasizes the need “to accelerate movement towards Universal Health Coverage” [6]. The policy should therefore support government strategies towards improving service and financial coverage for persons with MNS disorders.

Identification of core health system inputs and funding mechanisms that are a pre-requisite for improving service coverage and meeting the psychological health needs of the population in LAMICs has been a key objective of the recently concluded Emerging mental health systems in low- and middle-income countries (EMERALD) project [7]. This project was carried out in six LAMICs, namely: Ethiopia, Nigeria, South Africa, Uganda, India and Nepal. The health system financing dimension of the Emerald project comprised of a raft of related research activities along the pathway towards universal health coverage, including quantification of resource inputs needed to scale-up mental health services, assessment of the impact of mental illness on household welfare, and identification of sustainable health financing strategies in each of the participating countries. In this article, we specifically report on the potential strategies for sustainable mental health financing in Uganda.

In this article in particular, we present potential strategies for sustainable mental health financing in Uganda based on an analytical framework developed by the project.

## Methods

The study applied a mixed methods approach involving documents review/analysis as well as key informant interviews with selected stakeholders. Strategies for sustainably financing mental health care in Uganda were specifically generated through a streamlined, stepped approach developed by the Emerald project consortium members.

### Analytical framework for sustainable mental health financing

Key dimensions of Emerald’s sustainable financing framework included: assessment of disease burden of mental disorders, assessment of household level economic impact of mental disorders, analysis of the general health and mental health system, assessment of projected resource needs for scaling up mental disorders, assessment of current and projected macro-fiscal situation; and identification and selection of appropriate financing mechanisms.

Assessments of the public mental health burden, mental and general health sectors as well as the current and projected macro-fiscal situation were accomplished via a desk review of relevant government and international documentation that form part of this report. Assessment of projected resource needs for scaling up mental disorders, undertaken earlier in the project provided estimates for what it takes to scale up a set of interventions for priority mental disorders [8]. To estimate the household level economic impact of mental disorders, the project carried out a household survey in Kamuli district, in eastern Uganda. This provided estimates (also reported elsewhere) for the health and general expenditure as well as income patterns for households with persons with mental illness [9], enabling the household economic impact of mental illness to be deduced.

### Situational analysis of mental health financing in Uganda

A desk based situation analysis was carried out to get a realistic understanding of the strengths, weaknesses and opportunities for sustainable mental health financing. The situational analysis was conducted via review of national and international documentation. The review focused on three key domains: the disease burden, the health system, and the macro-fiscal situation of the country. The documents reviewed among others included: WHO mental health Atlas, National Policy on Mental, Neurological and Substance abuse disorders, Health Sector Strategic and Investment Plan, Government of Uganda budget estimates 2016/2017, Government of Uganda health financing strategy 2016 [10–12].

#### Disease burden assessment

Estimates for the burden of mental, neurological and substance abuse disorders in the country were compiled out of data from the World Health Organization-Global Health database and national documentation. Estimates of the burden of non-communicable diseases in general and more specifically disability adjusted life years (DALYs) and years lived with disability (YLD) due to MNS disorders were retrieved.

#### Health system assessment

The assessment of the health system was based on the WHO’s health systems framework [13]. The six blocks of the health system, namely: governance, health workforce, financing, service delivery, essential health technologies, and information systems were assessed. Specific attention was given to the situation of financing of the health sector as a whole and the mental health sector more specifically. In the assessment, we looked at how much funding the sector received, the primary sources of funding and the proportion of the health budget allocated to mental health. Estimates were retrieved from the World Health Organization-Global Health Expenditure Database (2005–2013) as well as national documents.

#### Macro-fiscal situation

To better understand the context within which the health system operates, more specifically health financing, the macro-fiscal situation of the country was assessed. Estimates for macro-fiscal indicators were derived from the World Bank’s development indicators database and government documentation. With these estimates, we examined the trend of the gross domestic product (GDP) and GDP per capita, unemployment and inflation rate, government revenue, expenditure, debt and budget deficit.

### Key informant interviews with health and financing expert stakeholders

Based on findings of the desk review, key informants in health and health financing were subsequently engaged in a discussion (in-depth interviews) on how mental health can be sustainably financed within the Ugandan context. Key informant interviews were conducted with a total of eight stakeholders, comprising state actors in health and finance as well as non-state actors. These key informants were selected purposively on the basis of their knowledge on this subject, and included stakeholders from different sectors and perspectives (see “Box 1”). The interviews were guided by a Mental Health Financing Diagnostic Tool developed by the Emerald project. The findings were summarized under three themes: (a) perceived challenges/constraints to increased public health financing, including for mental health, (b) criteria for improved public health financing, including mental health (c) options for increased financing for public health (including mental health) and sustainable financing mechanisms.

The interviews provided a platform for comparison and corroboration of findings from document analysis as well as involving the participation of stakeholders in the generation of strategies for sustainably financing mental health within the context of the country.

#### Matrix of key informants/stakeholder and their affiliation

**Table:** 

	Informant/expert	Affiliation
1	Acting Commissioner, Clinical services-Ministry of Health	State actor—health
2	Principal Medical Officer, Mental Health and Substance abuse—Ministry of Health	
3	Principal Health Economist—Ministry of Health	
4	Executive Director—Butabika National Referral and Teaching Mental Hospital	
5	Assistant Commissioner, Infrastructure and Social Service Department—Ministry of Finance, Planning and Economic Development	State actor—finance
6	Principal Economist (health desk), Infrastructure and Social Service Department—Ministry of Finance, Planning and Economic Development	
7	Director—Mental Health Uganda (NGO)	Non stateactor
8	Health Economist World Bank Uganda/Formerly Assistant Commissioner Planning, Ministry of Health	

### Identification of strategies for sustainably financing mental health

**Fig. 1:**
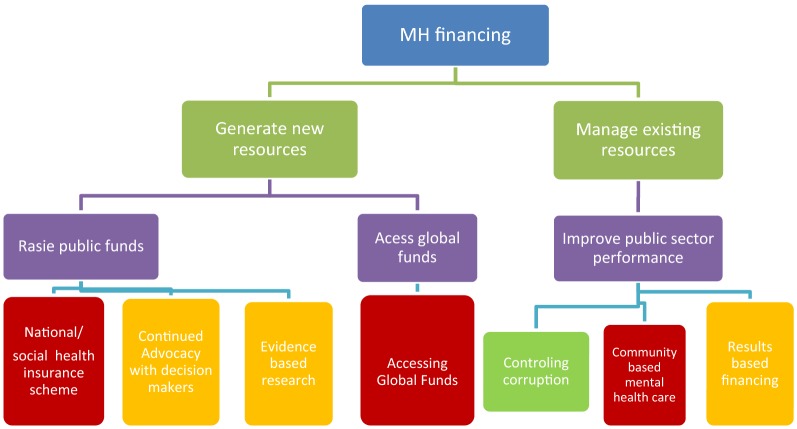
Emerald project’s conceptual framework for sustainable mental health financing. Emerald project’s mental health financing algorithm, adapted to the Ugandan context

Identification of strategies for sustainable mental health financing was based on findings from the situational analysis, as well as the resource need assessment, economic burden assessment and key informant interviews. Prioritization of strategies was facilitated by a mental health financing algorithm developed by Emerald project team (see Fig. 1). Under the algorithm, potential strategies were subjected to a set of criteria to determine those with greatest feasibility and utility. Selection criteria included: the potential for raising revenue (for health and mental health); potential for increased equity and financial/social protection; potential for stable and/or sustainable financing; and links to/integration with other programmes.

## Results

### Situational analysis of disease burden, health system development and macro-fiscal situation

#### Disease burden assessment

Uganda has an infant mortality ratio of 53 per 1000 live births, under five mortality ratio of 80 per 1000 live births and life expectancy at birth of 57 and 54 years for females and males respectively [14]. Findings revealed that while the disease burden in the country is still inclined towards infectious diseases, the prevalence of Non Communicable Diseases (NCDs) has rapidly increased over the past 10 years [15]; and comprised 13% of the national disease burden. While no national studies have been conducted to establish the prevalence of MNS disorders, available evidence indicates that these disorders are a growing public health challenge. According to the World Health Organisation Global Health Estimates for 2015, Mental Neurological and Substance abuse (MNS) disorders accounted for 5.2% of all Disability Adjusted Lives (DALYs) for Uganda [16]. Furthermore, 26.9% of years lived with disability (YLD) in Uganda are attributable to MNS disorders [16].

#### Analysis of the general health and mental health system

Health services in Uganda are delivered under a decentralized framework [10]. At the top, is the Ministry of Health, then local governments who are responsible for managing all health care providers under their jurisdiction. The Government of Uganda (GoU) health system is thus hierarchical, comprising of the National Referral Hospitals at the apex, below which are Regional Referral Hospitals. These provide specialized health services. Below these is the district health system which comprises of the district general hospitals, Health Centre IVs, Health Centre IIIs and Health Centre IIs; with variation in the staffing at each level as per the staffing norms. The lowest level (Health Centre I) is a satellite health facility with no definite physical structure, but rather an establishment comprising Community Health Workers (volunteers) serving as a link between the community and the formal health facilities. As regards the mental health system, there is a national referral mental hospital and mental health units in all the 13 regional referral hospitals. These provide outpatient and inpatient care [17]. Mental health care is provided at all levels of service delivery, including the general hospitals and health facilities as a component of the minimum health care package. The ratio of psychiatric unit beds per 100,000 population is 1.42 and 1.2 for mental hospital and general hospitals respectively [17]. The ratio of mental health care professionals to population is very low. The number of mental health care workers per 100,000 persons of the Ugandan population is estimated at 0.09 psychiatrists, 0.04 other medical doctors; 0.78 nurses; 0.1 psychologists; 0.01 social workers; 0.01 occupational therapists; 0.2 psychiatric clinical officers; and 6.4 psychiatric nurses [18, 19]. The major strength of the decentralized system of service delivery and integration of mental health into PHC is increased access to mental health care. However, prioritization and resource allocation have been noted to be still centralized, thereby affecting efficiency of service delivery.

#### Health system financing

The World Health Organization estimate for the per capita expenditure on health in Uganda is $52 [11]. The Total Health Expenditure (THE) as a percentage of GDP has stagnated at about 8.5% since 2011 dropping further to 6.9% in the financial year (FY) 2015/6 [20]. General Government Health Expenditure on health is 2% of the GDP and 25% of THE [11]. In the past, low priority has been given to Non Communicable Diseases (NCDs); NCDs comprised 3.8% of the Current Health Expenditure (CHE) in 2011/12. The trend is however changing positively as the current Health Sector Development Plan (HSDP) allocates 17% of the health budget to NCDs. Mental health expenditure alone accounts for 0.9% of the total health budget of the HSDP. The current HSDP estimates a total expenditure of US$144.93 million on mental health over its lifetime, i.e. 2015/2016–2019/2020 [21]. The largest proportion of this cost covers treatment (91%), prevention taking 8%, and other health services taking 1%. The HSDP (under which programme areas operate) allocates 54% of its total cost to health products and technologies, 16% to logistics management, 8% and 9% to health workforce and infrastructure respectively and 8% to service delivery systems. Health information accounts for only 0.2% of the total health cost.

The trends in the proportional contribution to health care by each financing source show that there is a heavy reliance on direct out-of-pocket expenses and external resources. The primary sources of health care financing in the country are; out of pocket expenditure (40%), donor funding (34%) and general government expenditure (26%) [11]. The health insurance sector in Uganda is still under-developed and contributes very little as a source of health financing, (estimated at 2%) [11]. The proposed national health insurance scheme (NHIS) has remained a plan awaiting government approval since 2007. The NHIS bill of 2012 provides for social, community and private commercial health insurance schemes, but only stipulates mandatory implementation for public servants and employers with five or more employees as the beneficiaries. Notably, psychiatric and neurological conditions are included among the packages provided under the bill.

#### Assessment of projected resource needs for scaling up mental health care

We earlier conducted an assessment of the resource needs required to scale up a range of specified interventions for MNS disorders and expected health gains at population level using the One Health Tool. Results of this estimation exercise indicated that the resource needs for scaling-up mental health services to meet the desired coverage goals are substantial compared with the current resource allocation, particularly if priority disorders and cost-effective intervention strategies are selected. For example the cost of delivering key interventions for psychosis, depression and epilepsy at existing treatment coverage was estimated at US$ 0.06–0.33 per capita of total population per year. Implementation of the package of care at target levels of coverage was expected to yield between 291 and 947 healthy life years per one million populations, representing a substantial health gain for the currently neglected and underserved sub-populations suffering from MNS disorders. Depression specifically accounted for the largest proportion of generated public health gain [8].

As regards the breakdown of costs between the different categories of resource need for the scale-up, essential psychotropic drugs absorb a large share of over all costs, taking as high as 45%, followed by ambulatory and outpatient care (about 32%), inpatient care with 16%.

#### Current and projected macro-fiscal assessment

Over the last 5 years to financial year 2016, the country’s economic growth rate averaged at 4.5% compared to the 7% achieved during the 1990s and early 2000s [12]. With the population increasing at a rate of at least 3% per annum through these decades, per capita income growth decelerated from a rate of 3.6% recorded in the decades of 1990s and 2002, to about 2%. In the FY 2016/7, the real annual economic growth rate was 3.9%, a contraction from the previous FYs [22]. Notably 2016 was a difficult economic year for most countries in the sub-Saharan region with the region experiencing an economic dipping of 1.4%, the lowest growth in two decades. A large proportion of the population relies on low-paying informal jobs in the agriculture sector [20]. Public debt is high and continues to rise, the debt as a percentage of revenues has risen by 54% since 2012 and is expected to exceed 250% by 2018 [23]. With the deficit on an upward trend, there is limited room for health budgetary expansions.

#### Key challenges and opportunities

The key informant interviews revealed several challenges for mental health financing, confirming limitations highlighted in the situation analysis. Mental health care is considered a specialized service, with the greatest share of mental health budget funds allocated to the national referral mental hospital. The hospital is mostly accessible to the urban population in view of its location and a relatively poor referral system. Community mental health care is almost non-existent, and the poorer sections of the population thus hardly have access to mental health care. Additionally, the political will is not very strong. Enactment of the revised mental health law has stalled, and the sector’s strategic plan has remained a draft for over 5 years; thereby hindering progress. While the population is growing at a rate of 3% per annum, economic growth continues on downward trend. Per capita income growth has decelerated to about 2%. In an effort to boost the economy, the government has prioritized sectors that have the potential to improve the economy; and the health sector is not among these. Thus, allocation of funds to the health sector as a proportion of GDP has taken a downward trend. Compared to its neighboring African countries, Uganda allocates less funds to the health sector (as a proportion of the GDP). However, the country has a designated mental health desk at the Ministry of Health headquarters; which has helped to push the mental health agenda forward. Furthermore, the country is planning to implement more reforms to improve financing in the health sector; such as the ongoing deliberations on implementation of Results Based Financing and National Health Insurance policy.

### Identification of sustainable financing mechanisms

#### Proposed mental health financing strategies

The potential strategies identified for sustainable mental health financing have been categorized into efficiency strategies, advocacy and accessing international funding. Strategies were prioritized based on their potential for raising revenue (for health and mental health), potential for increasing equity and financial protection, potential for stable and/or sustainable financing, feasibility and links to or integration with other programmes.

#### Efficiency strategies

The strongest proposed strategy for sustainable mental health financing is implementing the National Health Insurance Scheme. By mobilizing new resources through the pooling of finances, the NHIS was believed to possibly reduce the catastrophic out-of-pocket expenditure and lessen the high burden of financing the health sector that the government currently shoulders. The NHIS will pool resources from private sources, thereby promoting equity through cross-subsidies, and creation of large pools (once high coverage is attained). The scheme is expected to increase welfare gain in health care through financial risk protection. The scheme’s proposed contribution structure is proportional, but not progressive as individuals will contribute a standard proportion of income (4%), to be supplemented by an additional contribution of 4% by the employers, increasing the likelihood of sustainability, as long as individuals are in gainful employment. The envisaged contribution will cater for the paying member plus 4 dependants, and therefore the estimated average contribution is likely to be too low and inadequate to buy a meaningful package of care. Furthermore, the current NHIS design does not explicitly mention how the scheme will fit in with, or be integrated within the existing financing mechanisms.

In its current state, the latest version of NHIS bill (2012) does not stipulate mandatory contributions for the informal sector, and yet the poorest sections of the population are employed in this sector. They would therefore need to be planned for if they are to have financial protection. For the informal sector especially the rural population, a model similar to that implemented in neighbouring Rwanda represents a potential approach. In this model, communities would partner with a community health facility that serves their catchment area to access health services. Community members would make regular payments to the health facility after which they access a given set of services. For such a strategy to be most effective, it was proposed that mental health care is scaled up to community level. At a scale up per capita cost of $0.34 [23], provision of mental health services at community level would be very feasible and the most cost effective way of providing mental health to larger sections of the population under a well-planned out health insurance scheme.

The respondents further suggested Results Based Financing (RBF) as another viable option that would make more funds available for additional essential health services like mental health care, resulting in more efficient utilization of resources. This system has been piloted in Uganda and evaluation of RBF pilots such as Northern Uganda health or NU health (2011–2015), World Bank Study (2003–2005) and the CORDAID Pilot (2009–2015) has shown improved health service cost-effectiveness, making it a more feasible and sustainable option that can improve equity. This funding mechanism also fits in well and can easily be integrated within the existing funding framework. However, contextual issues like managing staff workload at increased levels of demand for services and how RBF would play out under NHIS need to be well planned out. Furthermore decentralizing services that can be provided at community health facilities would allow for the mental health budget to be spread out in a manner that allows for available funds to be shared among a larger section of the population. An example of the community based care programme is the mental health Gap Action Programme (mhGAP) implemented in community health facilities in Kamuli district by the Programme for Improving Mental health carE (PRIME) [24, 25]. The program built capacity of non-specialised health care workers to provide basic mental health care at the health facilities. A similar model could be replicated across the country and the national referral mental hospital left to manage severe cases.

#### Advocacy

High levels of stigma towards persons with mental ill health still exist even among policy/decision makers. Sadly, decision makers are often reshuffled or transferred quite frequently and it is very likely that those targeted by previous advocacy campaigns leave office. Furthermore, if decision makers do not appreciate the burden of mental health, they will not prioritise it when allocating funds. It was thus recommended that advocacy with these decision makers should be done regularly so as to maintain and/or advance mental health efforts. Additionally, stakeholders emphasized a need for research evidence that strengthens the business case for mental health care as planners consider value for money when distributing funds. Such evidence would give a strong case to advocacy campaigns. Research could be targeted at showing the societal costs attributable to mental disorders (including lost output/production), versus the much lower costs of intervention scale-up and the health and economic improvements that would flow or follow for this investment of resources.

#### Accessing funding from development partners

Funding from development partners was still largely considered a viable option. It emerged that government would easily increase financing to the sector after an initial expansion/scale up of mental health care by development partners, especially if the funding is tagged to government partnership/continuity. An example cited was the funding boost by the African Development Bank (ADB) that saw the national referral mental hospital rehabilitated and refurbished as well as construction of 13 state of the art psychiatric units in all existing regional referral hospitals; significantly increasing service capacity. Government then took over the provision of mental health services at these facilities. However, much as donor funding can make more funds available thereby increasing equity and access to services, it is not a viable option when it comes to sustainability as it tends to depend on donor interests and diplomatic relationship.

## Discussion

This analysis was the first of its kind in Uganda, focusing on mental health financing. Although the stakeholders interviewed were few, the assessment was thorough as we accessed and considered much vital information from relevant documents and interviewed key informants with substantial knowledge on the subject.

The findings from the situational analysis of the country’s health system indicate a relatively high burden of YLDs for MNS disorders, low mental health specialists to population ratio, high out pocket payments for health care, a declining government health expenditure on health as a percentage of the GDP and a poor performing economy. The declining government expenditure on health shows that Uganda is not on track to achieve the health related sustainable development goals (SDGs) and this should be a matter of concern, given the fact that Uganda was party to the 2001 Abuja declaration in which African Union countries set a target of allocating at least 15% of their annual budget to improve the health sector. Similarly, analysis revealed that mental disorders pose a negative economic impact on households. Within this context and in light of competing government priorities, one of the significant strategies for sustainably financing mental health in Uganda is by implementing the National Health Insurance scheme. Rolling out the scheme would reduce the catastrophic out of the pocket expenditure of persons with MNS disorders who happen to be poorer as well as relieving government of the high burden of financing the health sector that it currently shoulders. Importantly, according to the plan, before the informal sector and the poorest get on the insurance scheme, free government services will be improved to ensure that these categories also access an acceptable quality of services. The NHIS has however remained a proposal for a long time and is yet to be passed. With a largely informal economy, coupled by a weak tax collection system, implementation of the scheme is likely to face challenges. By design, the NHIS will mostly benefit persons in gainful employment, who constitute a smaller section of the population. For example it stipulates a monthly contribution/deduction of 4% of the employee’s salary plus a mandatory contribution by the employer of 4%; which is likely to face resistance. For the case of public servants, the 4% mandatory contribution by the employer is expected from government, which seems to pose a challenge in light of the several competing health and policy priorities, weak health governance system and lack of fiscal resources. The current inequalities in the distribution of health system inputs between rural and urban areas, different levels of care and geographic areas therefore pose a likely threat to successful implementation of the scheme; resulting in low coverage and challenges in effective management.

Therefore for the NHIS to be most effective in attaining financial protection for persons with MNS disorders, we recommend that the scheme includes mandatory contribution at community health facilities, as these serve the largest section of poorer population.

We also recommend that mental health care is scaled up to community health facilities to improve both service coverage and financial protection. Providing mental health care at community level under the NHIS would be a viable way for sustainably financing mental health care, in view of the fact that the major source of financing is contributions from the beneficiaries in gainful formal employment, who unfortunately constitute a smaller section of the population. The main limitation is the fact that by design, NHIS will not easily bring the informal sector on board. Financing by NHIS at this level could be complimented by Results Based Financing in the public health facilities. Furthermore, decentralisation of health services that can be provided at community health centres from the national referral mental hospital would allow for the mental health budget to be shared across a wider section of population. A funding boost from development partners linked to government continuation of the system would be the fastest way for scaling up mental health care at the community level.

Prioritization and advocacy were identified as key aspects for sustainable financing. In line with some earlier studies, it was noted during this analysis that low prioritization of mental health is still a major challenge. Apparently, prioritization seems to be well articulated at policy level as mental health is a component of the National Minimum Health Care Package. However, it seems to be a different approach when it comes to resource allocation [26]. It would therefore be crucial that prioritization is also reflected in resource allocation as well for sustainable mental health financing; and hence a need for continuous advocacy.

Donor funding was considered an option, and indeed mental health has greatly benefited from grants by external development partners over the past couple of years (especially for infrastructure, service development, capacity building and research). This is certainly the funding option that has driven the current developments for the mental health programme. For example the ADB funding (Support to Health Sector Strategic Plan Project) raised the mental health expenditure as a percentage of the overall health expenditure from less than 1% in 2001 to as high as 4% in 2005 [19]. The funding levels eventually dropped when donor funding ended, but remained reasonably higher than before. However, donor funding may not be a very reliable option for sustainable financing given the global trend of governments moving away from over reliance on external funding, which is dependent on donor interests and diplomatic relationship, and can therefore never be guaranteed. For example, the Uganda National budget contribution from development partners has continued to decline from as high as 30.6% in the 2008/2009 National budget to 24.3% in the 2017/2018 national budget.

In conclusion, the NHIS is a likely major health financing mechanism for raising additional resources for the sector. With mental health care scaled up under the NHIS, persons with MNS disorders will enjoy financial protection and improved health service coverage. The NHIS can therefore be considered a potential financing strategy in addition to Results Based Financing, which has been proven to be effective; and an alternative to out-of-pocket financing for mental health care.
